# Clinical Implications of Extracellular HMGA1 in Breast Cancer

**DOI:** 10.3390/ijms20235950

**Published:** 2019-11-26

**Authors:** Olga Méndez, José Pérez, Jesus Soberino, Fabricio Racca, Javier Cortés, Josep Villanueva

**Affiliations:** 1 Vall D’Hebron Institute of Oncology (VHIO), 08035 Barcelona, Spain; omendez@vhio.net (O.M.); jacortes@vhio.net (J.C.); 2 IOB Institute of Oncology, Quironsalud Group, 08023 Madrid & Barcelona, Spain; josemanuel.perez@quironsalud.es (J.P.); jesus.soberino@iob-onco.com (J.S.); docfabro@gmail.com (F.R.); 3 Medica Scientia Innovation Research (MedSIR), 08007 Barcelona, Spain; 4 Centro de Investigación Biomédica en Red de Cáncer (CIBERONC), 28029 Madrid, Spain

**Keywords:** high mobility group A1 (HMGA1), receptor for advanced glycation end products (RAGE), cancer secretome, triple-negative breast cancer (TNBC), tumor biomarkers

## Abstract

The unconventional secretion of proteins is generally caused by cellular stress. During the tumorigenesis, tumor cells experience high levels of stress, and the secretion of some theoretically intracellular proteins is activated. Once in the extracellular space, these proteins play different paracrine and autocrine roles and could represent a vulnerability of cancer. One of these proteins is the high mobility group A1 (HMGA1), which is frequently overexpressed in tumors and presents a low expression in normal adult tissues. We have recently described that HMGA1 establishes an autocrine loop in invasive triple-negative breast cancer (TNBC) cells. The secretion of HMGA1 and its binding to the receptor for advanced glycation end products (RAGE) mediates the migration, invasion, and metastasis of TNBC cells and predicts the onset of metastasis in these patients. In this review, we summarized different strategies to exploit the novel tumorigenic phenotype mediated by extracellular HMGA1. We envisioned future clinical applications where the association between its change in subcellular localization and breast cancer progression could be used to predict tumor aggressiveness and guide treatment decisions. Furthermore, we proposed that targeting extracellular HMGA1 as monotherapy using monoclonal antibodies, or in combination with chemotherapy and other targeted therapies, could bring new therapeutic options for TNBC patients.

## 1. Introduction

In the last decade, the profiling of the cancer secretome has been widely used as a tool for biomarker discovery projects, as well as for the study of different aspects of cancer biology and therapeutics [1,2,3,4]. The secretome is a sub-proteome made of secreted proteins, extracellular domains of plasma membrane receptors shed by proteolytic events, and different kinds of extracellular vesicles. Extracellular vesicles mainly participate in cell-to-cell paracrine communication, while soluble proteins in the secretome contribute to the autocrine and paracrine communication among tumor cells and between tumor and stromal cells [5]. More specifically, the cancer secretome contains different kinds of proteins, including extracellular matrix proteins, growth factors, proteases, cytokines, and chemokines. All these families of proteins have been associated with critical aspects of cancer progression, such as tumor invasion and metastasis [6,7,8,9]. 

The omics profiling in cancer research is generally performed by genomics and transcriptomics analysis. However, the cancer secretome is more accurately measured using proteomic techniques, considering that levels of messenger ribonucleic acid (mRNAs) and proteins codified by genes that give rise to the secretome have shown a poor correlation [10]. Our laboratory has focused methodologically on the characterization of the cancer secretome by quantitative proteomics and statistical modeling [11,12,13]. The comparative proteomic profiling of cancer cell line secretomes with different invasive properties resulted in the detection of a group of proteins that correlate strongly with the invasive potential of breast cancer tumor cells. Several of them were well-known proteases, growth factors, and extracellular matrix proteins, previously related to tumor migration, invasion, and metastasis. However, the careful analysis of the proteomic profiles showed that a large portion of the differentially secreted proteins was not secreted through the classical endoplasmic reticulum (ER)-Golgi secretory pathway, and most of them had a well-defined intracellular function [13,14]. Our group had already demonstrated that cancer cell line secretomes comprised of a group of non-classical secreted proteins that, although they did not follow the classical secretory pathway, were actively secreted from living cells [13,14].

We decided to study a nuclear protein called the high mobility group A1 (HMGA1) out of the group of non-classical secreted proteins since it was significantly over-secreted from invasive breast cancer cells. HMGA1 is a chromosomal protein that binds to the minor groove of Adenine-Thymidine (AT)-rich deoxyribonucleic acid (DNA) strands through its three AT-hooks and controls the transcriptional activity of several genes by interacting with the transcription machinery [15]. HMGA1 is expressed at high levels during embryonic development, but its expression is low or absent in adult differentiated tissues [16]. The re-expression of HMGA1 in adults has been associated with the onset of diseases, including diabetes and cancer. In breast cancer, the highest expression of HMGA1 corresponds to the triple-negative breast cancer (TNBC) subtype. TNBC accounts for approximately 10–15% of all diagnosed breast cancers. The majority of these tumors do not usually express hormone receptors and human epithelial growth factor receptor 2 (HER2), and this is why they are also known as TNBC. [17]. TNBC tumor subtype has a worse prognosis than luminal and HER2-positive breast cancer subtypes and is associated with a high risk of distant recurrence, mainly in the first two years after diagnosis [18]. This may result from its aggressive tumor biology, or rather it may be because this tumor subtype does not respond to either hormonal or anti-HER2 therapies.

Despite gene mutations or rearrangements are rare in malignant solid tumors, *HMGA1* is often overexpressed in tumor tissues, and this overexpression frequently correlates with the presence of metastasis and reduced patient survival [19,20,21,22,23]. Up to now, the proposed mechanisms for the HMGA1 role in tumorigenesis were related to its transcriptional regulation actions. Our recent publication, which describes an alternative mechanism by which extracellular HMGA1 mediates cancer migration, invasion, and metastasis in breast cancer, offers a new view on the role of HMGA1 in cancer. A thorough review of the HMGA1 literature in cancer research was out of the scope of this review; instead, we refer readers to the following excellent reviews on this topic [19,24,25,26,27,28,29]. In this review, we had first summarized the highlights of our recent work, and then we had hypothesized about the cancer diagnostic and therapeutic implications of the extracellular function of HMGA1 [30]. 

## 2. Extracellular HMGA1 Sheds New Light on the Role of HMGA1 in Cancer Biology

**Figure 1:**
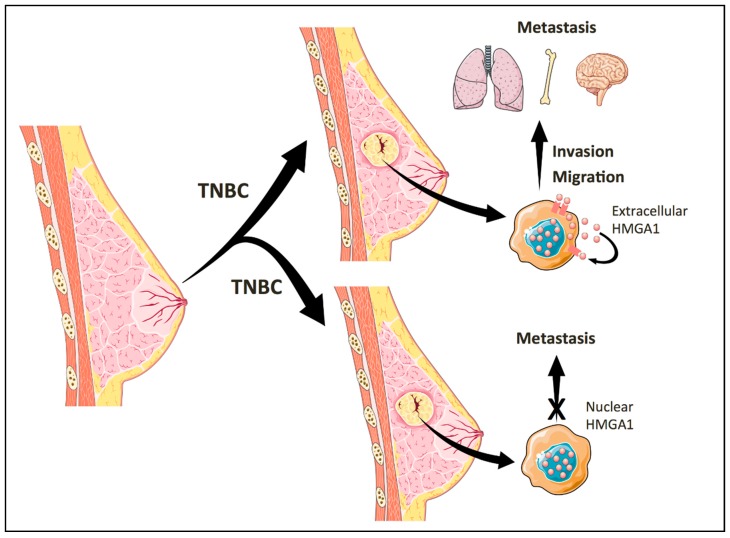
Role of extracellular HMGA1 (high mobility group A1) in triple-negative breast cancer (TNBC). The secretion of HMGA1 in TNBC cells increase their migratory and invasive phenotype and correlates with an increased incidence of distant metastasis in TNBC patients. TNBC tumors with nuclear HMGA1 show a decreased incidence of metastasis when they are compared to TNBC tumors with cytoplasmic HMGA1.

In the following section, we had focused on the most intriguing results of our work and how they could provide new opportunities to understand the role of HMGA1 in cancer biology. These results are summarized in Figure 1.

### 2.1. HMGA1 is Secreted by Invasive Breast Cancer Cells

The over-secretion of HMGA1 by invasive breast cancer cells opens the possibility that HMGA1 establishes new molecular interactions in the extracellular space that could complement its function as a transcriptional regulator in tumor cells. Another HMG protein, the high mobility group B1 (HMGB1), also previously known as HMG-1 and amphoterin, can also be secreted from both tumor and immune cells. In fact, HMGB1 can either be passively released or actively secreted from several cell types, including different immune and tumor cells [31,32,33]. While nuclear HMGB1 performs different functions related to gene transcription, DNA repair, and nucleosome structure maintenance, extracellular HMGB1 is a bona fide damage-associated molecular pattern (DAMP) [34]. DAMPs are a series of endogenous molecules with defined intracellular functions that are released to the extracellular space upon cell damage or stress through ER-Golgi independent pathways [35]. Once in the extracellular space, DAMPs promote the activation of pattern recognition receptors, including Toll-like receptors (TLRs) and receptor for advanced glycation end products (RAGE). The release of DAMPs activates the innate immune system, which results in host defense and tissue repair activities, as well as chronic inflammation in different diseases [35,36].

The secretion of HMGB1 can be triggered by different cellular insults that lead to cellular stresses and death, and it is also associated with cell migration [33,37]. Both HMGB1 and HMGA1 lack a signal peptide, and hence cannot enter into the classic ER-Golgi secretory pathway. In the case of HMGB1, its non-classical secretion seems to be mediated by secretory lysosome-mediated exocytosis [38]. Upon the trigger for secretion, HMGB1 is modified by different posttranscriptional modifications (PTMs), including acetylation, ADP-ribosylation, methylation, and phosphorylation [38,39]. In the case of HMGA1, a full secretion pathway has not been described. However, PTMs seem to mediate the secretion of HMGA1 since casein kinase 2 (CK2), a kinase that phosphorylates HMGA1 in vitro, is able to regulate its secretion by TNBC cells. Furthermore, conversely to HMGB1, HMGA1 is not released from apoptotic tumor cells [13], and its secretion clearly correlates with an invasive phenotype in TNBC cells [30].

The study by immunofluorescence of the HMGA1 expression and localization in breast cancer cells that secrete the protein led us to detect an accumulation of HMGA1 in the cytoplasm of these cells. Although the cytoplasmic localization was faint compared to its nuclear levels, we were able to show that the detection of the cytoplasmic localization of HMGA1 was a surrogate of its secretion. In a personal communication, Dr. Raymond Reeves, who pioneered the biochemical and molecular studies of HMGA1, brought to our attention that his laboratory had previously described a cytoplasmic localization of HMGA1. In these studies, the Reeves’ lab uncovered a dynamic, cell cycle-dependent translocation of HMGA1 proteins from the nucleus into the cytoplasm and mitochondria of NIH3T3 cells [40]. However, it is unclear whether the mitochondrial and cytoplasmic localization observed in those studies would be related to its secretion.

### 2.2. Extracellular HMGA1 is a Ligand of RAGE

RAGE is a member of the immunoglobulin receptor superfamily [41]. Under normal physiological conditions, RAGE expression is low in most adult cell types, except in the alveolar lung epithelium. However, RAGE is overexpressed in pathological conditions, such as inflammation, Alzheimer’s, diabetes, cardiovascular diseases, obesity, and cancer [42,43,44,45,46]. RAGE has multiple protein ligands, including the advanced glycation end-products (AGEs), proteins of the S100 family, fibrillar proteins, such as amyloid-beta, HMGB1, and now HMGA1 [30,47,48,49]. 

The binding of ligands to RAGE elicits cellular responses, activating different cellular signaling pathways, including nuclear factor kappa-light-chain-enhancer of activated B cells (NF-kB), mitogen-activated protein kinase (MAPK), and the Janus kinase (JAK)-signal transducer and activator of transcription (STAT) [41]. Several of the RAGE ligands, including HMGB1 and some of the S100 proteins, have been linked to tumorigenesis in in vitro studies, supporting the direct mediation of RAGE in the proliferation, migration, survival, and invasion of different tumor cells [37,50,51]. Furthermore, the binding of HMGB1 to RAGE has been associated with tumor growth and metastasis in a murine model [46]. However, the role of extracellular HMGB1 in cancer is complex since it can be actively or passively released from immune and tumor cells and bind to different receptors. In fact, HMGB1 can exert both pro- and anti-tumor responses [52,53]. 

In our recent report, we added HMGA1 to the list of ligands of RAGE [30]. We demonstrated both a physical interaction of HMGA1 and RAGE and a functional phenotype mediated by the binding of HMGA1 to RAGE in TNBC cells. The abrogation of the HMGA1-RAGE interaction significantly decreased the pro-migratory and invasive phenotype of TNBC cells. However, we could not rule out that HMGA1 also binds to other receptors, such as Toll-like receptors (TLRs) in tumor cells. The establishment of an HMGA1-RAGE pathway could help to clarify the complex array of cellular processes mediated by RAGE in tumor cells.

### 2.3. Extracellular HMGA1 Mediates Adhesion, Invasion, and Migration of Invasive Breast Cancer Cells

Mounting evidence accumulated for two decades has suggested an oncogenic role for HMGA1. Since the mid-nineties, various reports proposed that *HMGA1* is an oncogene, mediating neoplastic transformation [54,55,56,57]. Afterward, several groups have specifically linked HMGA1 to migratory and invasive phenotypes in different tumor cells. Various works have shown how different microRNAs modulate the migration and invasion of tumor cells by targeting HMGA1 [58,59,60]. It has also been proposed that HMGA1 can regulate the expression of matrix-metalloprotease 2 (MMP2), and hence tumor invasion. In addition, there are other molecular mechanisms involving HMGA1 that facilitate migration and invasion, including cyclooxygenase-2 (COX-2), the Wnt family of secreted glycolipoproteins via the transcription co-activator β-catenin (Wnt/β-catenin), the epithelial-to-mesenchymal transition (EMT), the transforming growth factor-beta (TGF-ß), and the plasminogen activation pathways [22,61,62,63,64,65]. Additionally, on a different note, HMGA1 has been recently found to play a role in intestinal homeostasis, which could impact intestinal tumorigenesis [66]. 

In our research, we demonstrated that the over-secretion of HMGA1 by invasive breast cancer cells could somehow mediate the invasive phenotype of these cells [30]. The first experiments using a series of antibodies against HMGA1 showed that the migration and invasion of different invasive breast cancer cells were significantly blocked. The striking results appeared when we added recombinant HMGA1 on cells in which the expression of HMGA1 was ablated, and we were able to partially recover the migratory and invasive phenotype of MDA-MB-231 TNBC cells. We do not know the reason for not obtaining a full phenotypic recovery. Perhaps, the nuclear HMGA1 was necessary for a complete mediation of the migratory and invasive phenotype mediated by HMGA1, or our recombinant HMGA1 produced in *Escherichia coli* was not fully functional due to the lack of PTMs. Nevertheless, our experiments proved that extracellular HMGA1 was able to mediate migration and invasion in tumor cells. 

Regarding the molecular mechanism by which extracellular HMGA1 mediates a pro-invasive phenotype, we demonstrated that extracellular HMGA1 regulated adhesion of TNBC cells. Upon identifying extracellular HMGA1 as a ligand of RAGE in TNBC cells, we determined that extracellular HMGA1 induced a decreased adhesive phenotype in invasive breast cancer cells. The *RAGE* gene is a close relative to other genes coding for adhesion receptors belonging to the cell adhesion molecules (CAMs) family [67]. 

Therefore, based on our results, we considered that an autocrine loop of HMGA1-RAGE was responsible for increased mobility of tumor cells, and therefore an enhanced migratory ability. We believed that the increased adhesion occurring when extracellular HMGA1 was blocked was, at least, partially responsible for the decreased migratory phenotype observed. However, a mechanistic explanation of how extracellular HMGA1 could impact tumor invasion is not yet known. We could not rule out that adhesion, migration, and invasion would need the coordinated effort of both the nuclear and the extracellular form of HMGA1. 

### 2.4. Extracellular HMGA1 Correlates With the Incidence of Metastasis 

In addition to the cell-autonomous processes described in Section 2.3, we confirmed that extracellular HMGA1 mediated metastasis in an orthotopic xenograft mouse model of TNBC. The expression of HMGA1 has been previously shown to promote and/or correlate with an increased incidence of metastasis by mechanisms, including the regulation of microRNAs, stemness, and the EMT [64,68,69]. 

In our experiments, both the ablation of HMGA1 expression and the blocking of the extracellular form with a specific antibody clearly decreased the incidence of metastasis in our xenograft model [30]. Although our results confirmed the impact of HMGA1 on metastasis, the fact that the ablation of HMGA1 expression and blocking the extracellular form of HMGA1 has similar effects on metastasis is at least puzzling. Based on our results, the extracellular form of HMGA1 would be largely responsible for the role of HMGA1 in metastasis. However, additional work using different experimental models and further mechanistic studies are needed to put our results in the context of previous research on HMGA1. Among HMG proteins, both HMGA2 and HMGB1 have been previously linked to metastasis. In the last few years, several works have described how different microRNAs regulate the development of metastasis by targeting HMGA2 [70,71,72]. Increasing evidence suggests that HMGB1 promotes metastasis in different cancer types by using different mechanisms. A recent paper demonstrated that extracellular HMGB1 could induce breast cancer metastasis by binding to TLR4 in a macrophage inhibitory factor (MIF)-dependent manner [73]. Another work described how an HMGB1-TLR4 interaction in platelets promoted metastasis in lung cancer [74]. TLR2 has been also reported to mediate the HMGB1 role in metastatic pancreatic cancer [75]. Additionally, the binding of HMGB1 to RAGE has similarly been described to promote metastasis in a Lewis lung carcinoma mouse model [46].

Together with the preclinical experiments, we were able to show a correlation between the subcellular localization of HMGA1 in primary tumors from TNBC patients and the incidence of distant metastasis [30]. In this case, we used the cytoplasmic localization of HMGA1 measured by immunofluorescence in tumors as a surrogate of secretion, based on our studies with cell lines in vitro (see Section 2.1). The expression of HMG proteins has been widely linked to tumor progression in different tumor types. Several reports have addressed the relationship between the overexpression of HMGB1 with the tumor progression in different cancer types, including lung, osteosarcoma, cholangiocarcinoma, colorectal, and liver cancer [76,77,78,79,80]. In the case of HMGA1, there is also some evidence that links its expression to tumor progression in different tumor types [21,81,82,83]. Our work went a step beyond and proposed that not only the overexpression but also the subcellular localization of HMGA1 could help as a tumor progression biomarker. However, more work is necessary since we had only observed this effect in TNBC and using exclusively immunofluorescence. This observation has potential clinical implications that have been discussed in the following Section.

## 3. Clinical Implications of Extracellular HMGA1

### 3.1. Preliminary Considerations

Ever since the discovery of the upregulation of HMGA1 in tumors, several reports have identified HMGA1 as a candidate tumor biomarker. Although *HMGA1* gene mutations are rarely identified in solid tumors, gene amplification is more frequent; consequently, HMGA1 protein is widely overexpressed in different tumor types, as previously mentioned in Section 2.

This protein overexpression in tumors, together with its low expression in normal adult tissues, makes HMGA1 an excellent target for drug development, as well as an ideal biomarker of tumor progression. However, its lack of enzymatic activity and the nuclear localization of this protein have seriously hampered the therapeutic options based on HMGA1. 

**Figure 2:**
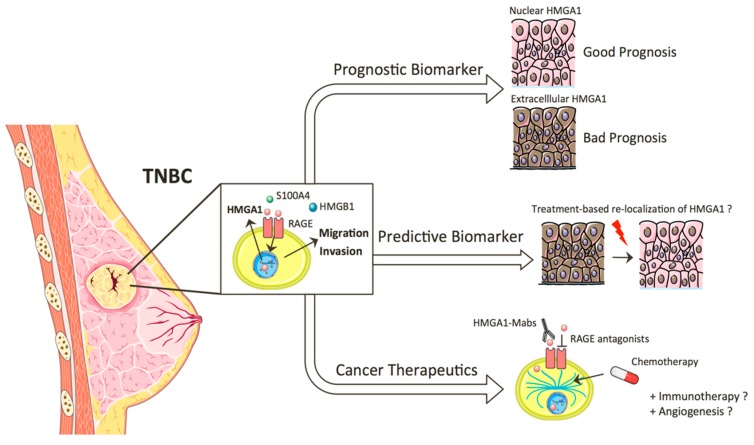
Clinical applications of extracellular HMGA1 in TNBC. The secretion of HMGA1 and the alteration of its subcellular localization in metastatic TNBC have potential diagnostic and therapeutic implications in the management of TNBC patients.

Fortunately, the novel tumorigenic phenotype mediated by HMGA1 in the extracellular space, in conjunction with the observed association between the change in subcellular localization of HMGA1 and breast cancer progression, opens a number of applications in a wide range of settings, including prognostic assessment, monitoring treatment response, and development of new anticancer therapies (Figure 2). 

### 3.2. Prognostic Biomarker

The change in the subcellular localization of several proteins is relevant for the outcome prediction in different tumor types. In this way, the subcellular localization of beta-catenin is one of the clearest examples of how the subcellular localization of a protein can be important for cancer prognosis. The E-cadherin/*β*-catenin complex at the plasma membrane sustains the integrity of epithelial tissue morphology [84]. The dysregulation of this protein complex during tumorigenesis induces the mislocalization of *β*-catenin, which is found in the cytoplasm and nucleus in a large number of tumors. In the nucleus, *β*-catenin becomes a transcription factor that plays a key role in the abnormal activation of the Wnt signaling pathway. The nuclear localization of *β*-catenin measured by immunostaining has been used for the diagnosis of endometrial cancer, and it has been associated with poor prognosis in different types of cancer, including colorectal, non-small cell lung, and hepatocellular carcinoma [85,86,87,88]. In a step closer to HMGA1, we found that the subcellular localization of S100 proteins could inform cancer outcomes. For example, the nuclear localization of S100A4 has been correlated with worse prognosis in ovarian, breast, and colorectal cancer [89,90,91]. Furthermore, the translocation of S100A4 and other S100 proteins to the nucleus in both stromal and tumor cells has been suggested to be mediated by their extracellular binding to receptors, such as RAGE [92]. Finally, the subcellular localization of HMGB1 has also been linked to cancer prognosis. In colorectal cancer, cytoplasmic HMGB1 in tumor cells is associated with poor outcome, while nuclear HMGB1 is present in both normal and tumor cells [93]. However, in other cases, an increased cytoplasmic HMGB1 correlates with a good prognosis [94]. This apparent contradiction could be explained by the different roles that this protein plays depending on cancer subtypes and/or disease stages. 

Regarding HMGA1, although our initial study only included a small number of patients, the subcellular localization of HMGA1 in primary triple-negative breast tumors seems to be significantly correlated with the development of distant metastasis [30]. Based on these results, an obvious clinical application of these findings would be the prognostic categorization of patients with this tumor subtype.

Therefore, it would be necessary to confirm that HMGA1 could predict tumor aggressiveness and also guide treatment decisions by measuring the protein levels and analyzing the subcellular localization of HMGA1 in the primary tumors of a large cohort of TNBC patients with and without metastatic involvement. Moreover, the evaluation of this function in other breast cancer subtypes and even in additional cancer types, including colorectal, pancreatic, and ovarian, in which HMGA1 is also highly expressed, merits additional investigation.

### 3.3. Predictive Biomarker

Different studies have reported that both HMG and S100 proteins could be used as biomarkers of therapeutic response in cancer patients. In the case of HMGB1, changes in the levels of circulating HMGB1 have been associated with the response to neoadjuvant therapy in breast cancer patients [95]. The overexpression of HMGA2 has also been linked to chemoresistance in colorectal cancer, although only in an in vitro model [96]. Moreover, the levels of nuclear HMGA2 have been correlated with shorter progression-free survival in metastatic bladder cancer patients receiving cisplatin [97]. Regarding the S100 family, different members, including S100A2, S100A8, and S100B, have been correlated with chemotherapy response in both breast and pancreatic cancer patients [98,99,100].

In breast cancer, the levels and subcellular localization of HMGA1 could be also useful to evaluate the antitumor efficacy and monitor the response to a specific therapy in TNBC patients. Based on these results, it is required to assess, either prospectively or retrospectively, the changes in the levels and/or subcellular localization of HMGA1 in TNBC patients treated with chemotherapy. 

This could be preferably performed using the neoadjuvant setting through the evaluation of the changes in the levels and/or subcellular localization of HMGA1 assessed by immunofluorescence between the pretreatment core biopsy and an on-treatment biopsy or the post-treatment surgical specimen. A change in the levels and/or subcellular localization of HMGA1 could help to identify those patients who derive more benefit from the neoadjuvant treatment. 

In the metastatic scenario, a setting more limited by the availability of paired tumor samples, it would be very appropriate to analyze the changes in the levels and/or subcellular localization of HMGA1 in circulating tumor cells (CTCs). The detection of changes in the subcellular localization and or the levels of HMGA1 in CTCs could offer the potential to more accurately predict patient outcomes than classical tumor response assessment methods, such as radiological evaluation or serum tumor markers. However, CTCs might not be detectable from all metastatic patients.

### 3.4. Therapeutic Implications of HMGA1 in TNBC

Due to the limited availability of pharmacological therapies and its aggressive clinical course, TNBC is the subtype with the worst outcome with a median overall survival of approximately 18 months or less. This result contrasts with the overall survival of patients with advanced luminal or HER2-positive breast cancer that ranges from 48 to 60 months.

Chemotherapy remains the mainstay of treatment for TNBC with disappointing results because despite the high sensitivity of early-stage TNBC to cytotoxic agents, in the metastatic setting, this treatment achieves relatively low rates of antitumor response ranging from 10% to 30% along with a short median progression-free survival that ranges from around six months in the first-line setting to approximately three months in refractory patients [101,102].

Thus, numerous efforts are currently being undertaken to develop more effective therapeutic strategies in order to improve the overall outcome of TNBC patients, and new advances in the knowledge of molecular biology of TNBC are increasing the treatment options of this complex disease.

HMGA1 has been a promising cancer drug candidate for years, but, being a nuclear protein, it was nearly impossible to target it. However, the novel pro-invasive and metastatic role of extracellular HMGA1, as well as the identification of the interaction between HMGA1 and RAGE, opens the possibility of targeting HMGA1, RAGE, and perhaps some of its other ligands as a new potential therapeutic option in patients with TNBC [30].

Unfortunately, there are no clinically viable therapeutics directly targeting *HMGA1* currently in clinical development. Nevertheless, several small molecule inhibitors of RAGE, binding either the extracellular or the intracellular RAGE domain, are being developed for the treatment of diabetic vascular complications and neurodegeneration, although the results obtained with these inhibitors are disappointing [103,104]. Even though RAGE inhibitors could represent a potential strategy for treating certain types of cancer and are available in clinical practice, no specific trials have evaluated the safety and efficacy of RAGE inhibitors in cancer patients either as monotherapy or in combination with chemotherapy and/or other targeted therapies to date.

Therefore, considering the new data regarding the oncogenic potential of the extracellular localization of HMGA1, along with other proposed functions for HMGA1, such as EMT activation and cancer stem cells (CSC) maintenance, new specific drugs targeting HMGA1 are needed. 

#### 3.4.1. Monotherapy against Extracellular HMGA1

##### Targeting Extracellular HMGA1

One of the main objectives of our work was to identify new therapeutic strategies to target the metastatic phenotype mediated by extracellular *HMGA1*. Although many studies have assessed the critical role of *HMGA1* in tumor progression, there has been relatively little work focused on targeting *HMGA1* in cancer considering the classical nuclear localization of this protein. 

Until now, disrupting the function of oncogenic transcription factors has been challenging, given the limitations of delivery and specificity. One exception is the recent case of targeting the *Myc* oncogene with a self-penetrating dominant-negative called Omomyc [105]. Even so, efforts to target critical downstream pathways of HMGA1 have shown some antitumor activity in preclinical models, such as COX-2 or STAT3 inhibitors [61,106]. 

Luckily, the novel pro-invasive and metastatic role of extracellular HMGA1 raises exciting opportunities to develop new compounds targeting extracellular HMGA1, including blocking monoclonal antibodies targeted against HMGA1 or soluble decoy receptors that would prevent HMGA1 from binding to its receptors. In fact, our laboratory is currently developing monoclonal antibodies directed against the extracellular form of HMGA1. Despite most monoclonal antibodies used in cancer therapy generally target membrane receptors, in some cases, monoclonal antibodies have also been successfully developed against ligands instead or in addition to its receptors. An example of the targeting of protein ligands in oncology is that of the vascular endothelial growth factor (VEGF). Several agents directed against the VEGF/VEGF receptor (VEGFR) signaling pathway have been developed and are now approved across several indications, although the first targeted antiangiogenic drug authorized for use in cancer patients was bevacizumab, a humanized monoclonal antibody that targets VEGF-A to prevent its interaction with VEGFR-1 and -2 [107].

##### Targeting RAGE

The full-length human RAGE consists of an extracellular (amino acid residues 23–342), hydrophobic transmembrane (residues 343–363), and cytoplasmic domains (residues 364–404). The extracellular structure of RAGE can be further subdivided into three immunoglobulin-like domains: a variable domain (residues 23–116) and two constant C1 (residues 124–221) and C2 (residues 227–317) domains [108]. 

In addition to the previously described oncogenic effects of extracellular HMGA1, RAGE expression is also higher in metastatic TNBC cell lines and is associated with poor outcome, playing an essential role in promoting breast cancer growth and metastasis [50,109,110], enhancing cell mesenchymal properties, and inducing EMT [111]. In this way, the blockade of RAGE could inhibit tumor progression and metastasis, and previous studies demonstrated that RAGE knockdown by small interfering RNA significantly decreased the tumorigenic potential of MDA-MB231 TNBC cell line [112].

Following the overexpression of RAGE in neurodegenerative disorders, such as Alzheimer’s disease, various antagonists binding to the extracellular or intracellular RAGE domain have been developed and evaluated in this context. Unfortunately, phase III clinical trials using the RAGE antagonist, Azeliragon, failed to meet their primary endpoint [104]. Despite the failure of targeting RAGE in Alzheimer’s, RAGE antagonists could still be valid as cancer drugs. However, due to its large number of ligands, the targeting of RAGE in cancer is unpredictable. Therefore, the results of this targeting in different cancer types could be completely different depending on the nature of the ligands expressed in each tissue. 

##### Clinical Approach to Design Single-Agent Studies

At the moment, the design of single-agent clinical studies based on the inhibition of RAGE and extracellular HMGA1 activity might be premature. However, in this section, we looked at the future and proposed a rationale to bring these candidates drug targets to the clinic, provided that sufficient experimental data is presented in the near future.

In the case of an HMGA1 inhibitor, the most reasonable proposal in the future for these compounds would be to design a phase Ib dose-escalation trial. The purpose of this study would be to evaluate the safety (maximum tolerated dose and dose-limiting toxicities) and preliminary efficacy of a specific HMGA1 inhibitor in patients with pretreated metastatic TNBC. Regarding RAGE inhibitors, and taking into account that these drugs have already been assessed in other diseases, it seems more appropriate to directly launch a phase II single-arm trial in pretreated patients with metastatic TNBC using Simon’s two-stage design, where overall response rate would be the primary endpoint. 

Another chance to clarify the mechanism of action of RAGE and HMGA1 inhibitors would be through the design of a phase 0 (window of opportunity) neoadjuvant trial, including patients with localized TNBC treated with a short-course administration of a RAGE or a future HMGA1 inhibitor. This study would represent the best opportunity to elucidate how these compounds work and also to explain the potential antitumor activity of these agents. 

The primary objective of this trial would be to test if the treatment with a RAGE or an HMGA1 inhibitor is able to change the subcellular localization of HMGA1 between the pretreatment core biopsy and the post-treatment surgical specimen. This study would also allow assessing the pharmacokinetics, pharmacodynamics, reduction in Ki67 proliferation index, and the discovery of other potential biomarkers.

#### 3.4.2. Combination with Chemotherapy

To our knowledge, the only evidence supporting a synergistic effect of the combination of chemotherapy and RAGE inhibitors comes from a preclinical model of pancreatic cancer. In this model, the combination of RAGE inhibitors with gemcitabine significantly reduced tumor growth compared with either agent alone [113]. So far, there is no data supporting the antitumor activity of the combination of chemotherapy and HMGA1 inhibitors.

In TNBC, chemotherapy, alone or in combination with targeted therapies, remains the standard of care [114]. This tumor subtype is characterized by the presence of cancer cells with mesenchymal features, confirming that EMT plays a major role in the progression of TNBC. The EMT program has also been implicated in chemoresistance, tumor recurrence, and induction of CSC properties [115,116]. Eribulin is a synthetic analog of the marine-sponge natural product halichondrin B with potent antiproliferative activities against a variety of human cancer cell types in vitro and in vivo through its antimitotic activity and other non-mitotic effects, such as reversal of EMT, resulting in a reduction of the capacity to metastasize in experimental models [117]. Eribulin is currently approved for the treatment of patients with locally advanced or metastatic breast cancer who have progressed after at least one chemotherapeutic regimen for advanced disease. The use of eribulin, in combination with other therapies, is being widely explored because its favorable safety profile makes it an ideal combination-treatment partner. 

Among others, HMGA1 and RAGE promote EMT in TNBC cell lines [68,111], and therefore, it would be of interest to evaluate the safety and efficacy of the combination of eribulin and a RAGE or a future HMGA1 inhibitor in patients with metastatic TNBC. Additionally, the combination of RAGE or HMGA1 inhibitors with other chemotherapy drugs frequently used in TNBC, such as carboplatin, taxanes, anthracyclines, or capecitabine, could also be explored.

#### 3.4.3. Combination with Targeted Therapies

##### Immunotherapy

Multiple lines of preclinical and clinical evidence have demonstrated that tumors can evade destruction by the immune system by expressing surface ligands that engage inhibitory receptors on tumor-specific T cells and induce immune tolerance [118]. Immune-related signatures have been abundantly found within TNBC, more specifically in the immunomodulatory subtype [119].

Early results of immunotherapy with immune checkpoint inhibitors in patients with metastatic TNBC have demonstrated encouraging activity with durable responses and substantial overall survival in pretreated patients. Unfortunately, there is a relatively small subset of patients that derive a benefit from single-agent treatment [120,121]. However, the increasing evidence in other cancers that combination with chemotherapy could improve the anti-tumor activity of programmed cell death ligand 1 (PD-L1)-targeted therapy led to the evaluation of the combination of chemotherapy and immunotherapy in patients with metastatic TNBC in the recently completed randomized phase III IMpassion130 trial with striking results. This study enrolled a total of 902 patients with untreated metastatic TNBC who were randomized to nab-paclitaxel plus atezolizumab or nab-paclitaxel plus placebo [101]. The addition of atezolizumab significantly increased median progression-free survival and overall survival, exclusively among patients with PD-L1–positive tumors. 

PD-L1 expression has been correlated with the expression of CSC markers and HMGA1 in clinical colorectal cancer specimens. Therefore, PD-L1 could participate in the maintenance of CSC self-renewal by activating HMGA1-dependent signaling pathways [122] Unfortunately, no data regarding this interaction has been reported in breast cancer. However, it also seems interesting to assess the efficacy and safety of the addition of a RAGE or a future HMGA1 inhibitor to the combination of chemotherapy and a PD-L1 inhibitor in patients with metastatic PD-L1-positive TNBC.

##### Angiogenesis

Angiogenesis plays an important role in cancer progression. The development of new vessels is decisive for the growth and spread of tumor cells because malignant tumors are unable to grow beyond a few millimeters without new vasculature [123]. Linderholm and colleagues analyzed the relationship between intratumoral VEGF levels and outcome in patients with TNBC cancer and non-TNBC. Compared with non-TNBC, patients with TNBC had significantly higher VEGF levels and worse survival [124]. Among the various antiangiogenic drugs, bevacizumab is the most widely studied in patients with breast cancer. To date, five phase III clinical trials have evaluated the addition of bevacizumab to chemotherapy in the first- and second-line treatment of patients with metastatic breast cancer, showing that the addition of bevacizumab resulted in statistically significant improvements in progression-free survival, the primary endpoint of these studies. Cancer subtype analysis demonstrated that TNBC patients achieved a greater benefit than hormone receptor-positive tumors [125,126].

Recently, it has been postulated that HMGA1 increases breast cancer aggressiveness by favoring tumor angiogenesis [127]. Therefore, the chance to target the HMGA1-RAGE axis in combination with anti-angiogenic and chemotherapy could represent an attractive therapeutic option in patients with metastatic TNBC, mainly with the combination of paclitaxel, bevacizumab, and a RAGE inhibitor or a future HMGA1 inhibitor.

## 4. Conclusions and Future Directions

Tumor cells have to cope with substantial levels of cellular stress, including hypoxia, nutrient starvation, and acidosis, in order to successfully generate tumors and promote metastasis. Growing evidence suggests that cellular stress is associated with the unconventional secretion of proteins, highlighting among them the secretion of DAMPs, such as HMGB1 and S100s proteins, that probably connects unconventional protein secretion with cancer. 

Based on our recent results, although HMGA1 binds to RAGE like other DAMPs, the secretion of HMGA1 does not fully fit with the concept of DAMP, considering that we have only shown a cell-autonomous phenotype. In fact, our results demonstrated that an HMGA1 autocrine loop was established in invasive TNBC cells, mediating a migratory and an invasive phenotype. However, we could not discard a danger signal triggered by HMGA1 in the tumor microenvironment, and future studies are needed to determine whether extracellular HMGA1 binds to other receptors beyond RAGE either in tumor or stromal cells.

The clinical significance of extracellular HMGA1 has yet to be understood, but our preliminary observations warrant further evaluation in different directions despite the important gaps in the knowledge of the putative role of extracellular HMGA1 and whether targeting this secreted form would benefit cancer patients. 

First of all, although HMGA1 is mostly nuclear in luminal breast cancer cells, it would not be unexpected that HER2-positive cells also secrete HMGA1. In addition, other cancer types in which the expression of HMGA1 is high, including colorectal, pancreatic, and ovarian cancer, could also secrete HMGA1. Nevertheless, if the secretion of HMGA1 in these cancer types is associated with tumor invasion, the onset of metastasis remains to be defined. 

Secondly, the alternative functions of non-classical secreted proteins by tumor cells might offer a new strategy to fight against cancer. This is especially relevant, taking into account that these proteins are not likely to be constitutively secreted by healthy tissues. Therefore, the use of monoclonal antibodies that specifically target the extracellular function of these multicompartment proteins might help to interfere with different aspects of tumorigenesis with a favorable toxicity profile. It seems particularly appropriate to combine chemotherapy or other targeted therapies, such as immunotherapy and antiangiogenics agents with HMGA1 inhibitors, in clinical practice.

Lastly, the evaluation of HMGA1 as a new prognostic biomarker in patients with TNBC, predicting the development of distant metastasis, merits additional investigation.
